# Supplementary material for: Barriers, Enablers, and Perceptions on Dietary Salt Reduction in the Out-of-Home Sectors: A Scoping Review
Source: Int J Environ Res Public Health. 2021 Jul 30;18(15):8099. doi: 10.3390/ijerph18158099 (PMC8345971; doi:10.3390/ijerph18158099)
Supplement: Supplementary file 1 [file ijerph-18-08099-s001.zip › ijerph-1235176-supplementary.pdf]

Table S1. Barriers, enablers, and perceptions on dietary salt reduction in the out-of-home sectors

Study (Country)	Findings														
	Perception			Barriers					Enablers						
	High salt is bad for health	Low salt is not tasty	Individual or organizational characteristic	Menu variability	Technical expertise for reformulation	Technical skills for cooking	Profit loss	Others	Training	Salt substitute	Gradual reduction of salt	Intervention	Food analysis studies or salt intake measurement	Others
Gase et al. 2011 (USA) [1]				costs and unavailability of low-sodium foods		unique features among food service settings		complexity of food service arrangements, lack of consumer demand for low-sodium foods, undesirable taste of low-sodium foods, preference for prepackaged products, lack of knowledge and experience in performing sodium standards, and existing multiyear contracts that are difficult to change.						

Petra et al. 2012 (Europe) [2]	99% of the respondents were aware which foods are low or high in salt.	90% of the participants added salt to dishes to enhance flavour and improve taste.							
Jaworowska et al 2012 (UK) [3]			Popular hot takeaway meals have alarming high salt content						
Coxson et al,2013 (US) [4]						A gradual reduction in dietary sodium	Range of proposed public health intervention		
Cotter et al,2013 (Portugal) [5]					Using herbs and spices		Weekly lessons on danger of high salt intake, (theory), lessons and practical in school garden of planting and collection of herbs for salt substitution at home	24 hour Urinary sodium excretion analysis	Blood pressure measurement
Lima et al 2013 (Brazil) [6]							Feasible dietary approach		
Maalouf et al 2013 (US) [7]				No comprehensive sodium content data in restaurant foods					
Antoniolli et al. 2013 (Australia) [8]								Sodium and saturated fat content were calculated from company websites	Nutritionally promoted fast foods may contain less sodium when selected

Frantz et al. 2013 (Brazil) [9]	-		9 stages of the Controlling Salt and Sodium use During Meal Preparatio n	
Christoforou et al 2013 (Australia) [10]		Failure of voluntary industry efforts suggest a regulated approach for ready meal products		Reformulation
Wong et al 2013 (Canada) [11]				Sodium claim And active education program of sodium reduced cooking At worksite, cafeteria
Johnston et al. 2014 (USA) [12]				A social marketing approach was used to educate consumers about the hidden sources of dietary sodium
Webster et al 2014 (Global) [13]				Legislation

Kim et al. 2014 (Korea and USA) [14]	Consumers' knowledge of the relationship between diets high in sodium and an increased risk of developing previously reported sodium-related diseases	Current consumer knowledge on the sodium content in food products was high						
Losby et al,2014 (New York) [15]		Understanding the complexity of the melas system for older adults	Engaging experts		Gradual and voluntary reduction of sodium content	Product substitution, recipe modification	Sodium nutrient analysis	Identifying partners with shared experience and common goals Working towards sustainable sodium reduction
Ma,G,X et al (2014) Philadelphia.US [16]	Lack of knowledge on the danger of high salt intake			Training in food preparation,procurement and marketing				Customer demand maintained, strategies and support
Mezue et al 2014 (Nigeria) [17]	Plan for a population-wide salt reduction strategy							
Marakis et al 2014 (Greece) [18]		Women added more salt than men in cooking						

Korosec et al 2014 (Slovenia) [19]						Market leaders have lower salt content	
Vanderlee et al 2014 (Canada) [20]					Positive impact of menu labelling		
Sookram et al. 2015 (Africa) [21]							overview of WHO supported interventions on salt intake reduction among Member States of the African
Enkhtungalag et al 2015 (Mongolia) [22]	Mongolia has one of the highest rates of stroke	Similar lifestyle			Pinch Salt intervention to reduce salt consumption among factory workers	Salt in tea contribute 30% of daily salt intake	
Kloss et al. 2015 (Europe) [23]			food safety issues, consumer acceptance concerns, cost concerns and complications arising from the use of sodium alternatives	Limited motivation among food manufacturers,	Food reformulation efforts have been made in the bread, meat, dairy and convenience foods industries.		

Trieu et al 2015 (Australia) [24]		• Reformulation with sodium content targets<br>• Consumer education<br>• Front-of-pack labelling<br>• Taxation of high-salt foods<br>• Public institution<br>Rigorous evaluation	
Lee et al. 2015 (South Korea) [25]	Prefer reduced sodium meals		
Webster et al 2015 (Australia) [26]		Drop Salt Campaign by NGOs and food industry to advocate the government to develop a national strategy to reduce salt and<br>Food reformulation<br>Health Star rating front pack labelling	
Hendriksen et al. 2015 (Netherlands) [27]			reduction of salt intake to 5 grams per day is expected to substantially reduce the burden of cardiovascular disease and mortality in several European countries.

Hendriksen et al. (2015) (Netherlands) [28]				Modification of food composition or by alteration of behavior may substantially reduce the median sodium intake using two scenarios from National Food Consumption Survey 2011.
Do et al,2016 (Vietnam) [29]			COMBI intervention- effective in lowering salt intake and improve knowledge and behaviours	
Land et al 2016 (Australia) [30]	Good knowledge	Use salt substitutes	Read labels	
Takada et al. 2016 (Japan) [31]			measure the difference in estimated daily salt intake by spot urine sampling of housewives and their family members 2 months after intervention between the groups.	

Regan et al. 2016 (Ireland) [32]	A series of multiple regression revealed that individual attitudes and beliefs related to health and salt were stronger predictors of support than sociodemographic factors , lifestyle or knowledge					
Quilez et al 2016 (Spain) [33]	Good acceptance of the reduced salt breads			Replacing salt with potassium citrate in bread		
Lee & Park 2016 (Korea) [34]	Most of the participants had relatively high levels of perception regarding the importance of sodium reduction	providing sodium-reduced meals were use of processed foods	limited methods of sodium-reduced cooking in worksite cafeteria			
McLaren et al. 2016 [35]					Population level intervention	Multicomponent intervention, Incoporate product reformulation and among men

Curtis et al.2016 (USA) [36]				Industry slow progress			National target setting (voluntary target)			Monitoring through Partnership of local and , state and national nhealth organizations
Park et al. 2016 (Korea) [37]		lack of various delicious low-sodium menus	Environmental factor such as pressure to maintain profit margins		Skill and techniques related to measuring sodium content and preparing RSM were emphasized by the interviewees.					key stakeholders' psychosocial characteristics and environment factors
Wang et al. 2016 (China) [38]								The CVD Policy Model -Population wide dietary salt reduction policies		
Van Buren et al. 2016 (Netherlands)[39]						Replacement of sodium chloride by potassium chloride, particularly in key contributing product groups,				
Grimes et al. 2017 (Australia) [40]	83% percent believed that Australians eat too much salt.									
Brooks et al 2017 (Boston, US) [41]								Increase availability of lower-sodium products		
Johnson et al. 2017 [42]						Use of low sodium salt sustitute	Specific sodium target	Behavior change intervention	Spot or 24 hours urinary sodium salt intake measurement	

Nilson et al. 2017 (Brazil) [43]					Monitoring sodium content of food		
Pravst et al 2017 (Slovenia) [44]						Sales-weighted sodium content	
Okuda,M et al (2017) (Japan) [45]					Home environment and salt –use behaviour intervention in secondary school .		
Harnack et al. 2017 (USA) [46]	Sodium added to food outside the home accounted for ≈70% of dietary sodium intake.						
Inguglia , E.S et al (2017) [47]			Microbial safety in low sodium meat products m	The use of salt substitutes , MSG or yeast extract			High presurre processing and power ultrasound
Hendriksen et al. (2017) (Netherlands) [48]							Different health impact model assessment from seven population health impact models may affect the health impact estimate.

Temme et al. 2017 (Netherlands) [49]							The salt content of bread, certain sauces, soups, potato crisps, processed legumes and vegetables have been reduced over the period 2011–2016 in Netherlands.	
Lacey,M et al (2018) (Canada) [50]	37 % believe that patient/resident would decrese with sodium reduction				Lower sodium foods, Increased availability of pre – packaged lower sodium products			Group purchasing organizations, Goverment prioritizing and providing suppose and resources, Improved tastes of lower soium foods

Gupta et al. 2018 (India) [51]			The lack of proper implementation of even existing food policy	decreased sales due to salt reduction.	social and cultural beliefs, a large unorganized food retail sector		The development and adoption of the National Multi-sectoral Action Plan	Most of the stakeholders were in alignment with the need for a salt reduction programme in India to prevent and control hypertension and related CVD.
Trieu et al. 2018 (Australia) [52]	the proportion who understood the adverse effects of salt (+9.0%, P=0.049)	A total of 73% reported that they had heard or seen the salt reduction messages.						
Levi et al. 2018 (Australia) [53]						A 6% reduction in sodium levels in soups overall was found from 2011 to 2014		

Zhang et al. 2018 (China) [54]					1.AIS – Application Based intervention Study using mobile application to reinforce and maintain lower salt intake<br><br>2. RIS (Restaurant based Intervention Study_ for consumers,cooks and res. Manager<br><br>3.HIS (Housewife intervention study_ for family chef<br>4. CIS (n Comprehensive Intervention study for evaluating all interventions	
Ahn et al. 2019 (Korea) [55]	Kn0wledge edge of the recommendation of salt , difference between sodium and salt	The majority (82%) was willing to reduce sodium in restaurant foods under the support of local government and they desired the promotion of participating restaurants and education on cooking skills to reduce sodium.	Maintaining taste and hindering the cooking process were the main barriers to reduce sodium use.	Purchasing experience		Supportive social enviroment , improving dietary habits of eating high salt foods

Sparks et al. 2019 (Vanuatu) [56]	total of 83% of participants agreed that too much salt could cause health problem	more than two-thirds of the population reported always/often adding salt to food during cooking/meal preparation and at the table, and always/often consuming processed foods high in salt				
Dodd et al 2019(Australia) [57]			Economic assessments of salt reduction efforts			
Beer-borst et al. 2019 (Switzerland) [58]					workplace program of nutrition intervention for employees and catering staff is feasible.	
Arcand et al 2019 (Latin America) [59]						Sodium content in packed foods
Yang et al 2019 (China) [60]				Stages of salt reduction in food industry	Vigorous advancements of salt reduction actions	

Park et al. 2020 (Korea) [61]			South Korea implemented its National Plan to Reduce Sodium Intake, with a goal of reducing population sodium consumption by 20%, to 3900 mg/day, by 2020.	
Bolton et al. 2020 (Australia) [62]	One-third (32 %) of participants reported adding salt at the table and 61 % added salt whilst cooking.			
Dunford et al 2020 (US) [63]		Packed food sources contribute 67% of sodium intake		
Sloan et al 2020 (US) [64]				Laws: labels, workplace, vending machines
Doggui et al 2020 (Eastern Mediterranean Region) [65]				Mandatory regulatory measures for universal salt iodization.

References

1. Gase, L.N.; Kuo, T.; Dunet, D.O.; Simon, P.A. Facilitators and barriers to implementing a local policy to reduce sodium consumption in the County of Los Angeles government, California, 2009. *Preventing chronic disease* **2011**, *8*, A33.
2. Mallia, P.; Gauci, C. The use of salt in restaurants. *Malta Medical Journal* **2012**, *24*, 29-33.
3. Jaworowska, A.; Blackham, T.; Stevenson, L.; Davies, I.G. Determination of salt content in hot takeaway meals in the United Kingdom. *Appetite* **2012**, *59*, 517-522, doi:10.1016/j.appet.2012.06.018.
4. Coxson, P.G.; Cook, N.R.; Joffres, M.; Hong, Y.; Orenstein, D.; Schmidt, S.M.; Bibbins-Domingo, K. Mortality benefits from US population-wide reduction in sodium consumption: projections from 3 modeling approaches. *Hypertension (Dallas, Tex. : 1979)* **2013**, *61*, 564-570, doi:10.1161/hypertensionaha.111.201293.
5. Cotter, J.; Cotter, M.J.; Oliveira, P.; Cunha, P.; Polónia, J. Salt intake in children 10-12 years old and its modification by active working practices in a school garden. *J Hypertens* **2013**, *31*, 1966-1971, doi:10.1097/HJH.0b013e328363572f.
6. Lima, S.T.; da Silva Nalin de Souza, B.; França, A.K.; Salgado Filho, N.; Sichieri, R. Dietary approach to hypertension based on low glycaemic index and principles of DASH (Dietary Approaches to Stop Hypertension): a randomised trial in a primary care service. *The British journal of nutrition* **2013**, *110*, 1472-1479, doi:10.1017/s0007114513000718.
7. Maalouf, J.; Cogswell, M.E.; Gunn, J.P.; Curtis, C.J.; Rhodes, D.; Hoy, K.; Pehrsson, P.; Nickle, M.; Merritt, R. Monitoring the sodium content of restaurant foods: public health challenges and opportunities. *American journal of public health* **2013**, *103*, e21-e30, doi:10.2105/AJPH.2013.301442.
8. Antoniolli, R.E.; Atkinson, L.F.; Palmer, M.A. Total lunchtime fast food purchases were lower in sodium and saturated fat when nutritionally promoted fast foods were ordered instead of traditional fast foods: A pilot study. *Nutrition & Dietetics* **2014**, *71*, 41-45, doi:10.1111/1747-0080.12044.
9. Frantz, C.B.; Veiros, M.B.; Proença, R.P.d.C.; Sousa, A.A.d. Development of a method for controlling salt and sodium use during meal preparation for food services. *Revista de Nutrição* **2013**, *26*, 75-87.
10. Christoforou, A.K.; Dunford, E.K.; Neal, B.C. Changes in the sodium content of Australian ready meals between 2008 and 2011. *Asia Pacific journal of clinical nutrition* **2013**, *22*, 138-143, doi:10.6133/apjcn.2013.22.1.10.
11. Wong, C.L.; Arcand, J.; Mendoza, J.; Henson, S.J.; Qi, Y.; Lou, W.; L'Abbé, M.R. Consumer attitudes and understanding of low-sodium claims on food: an analysis of healthy and hypertensive individuals. *Am J Clin Nutr* **2013**, *97*, 1288-1298, doi:10.3945/ajcn.112.052910.
12. Johnston, Y.A.; McFadden, M.; Lamphere, M.; Buch, K.; Stark, B.; Salton, J.L. Working with grocers to reduce dietary sodium: lessons learned from the Broome County Sodium Reduction in Communities pilot project. *Journal of public health management and practice : JPHMP* **2014**, *20*, S54-58, doi:10.1097/PHH.0b013e3182a0b91a.
13. Webster, J.; Trieu, K.; Dunford, E.; Hawkes, C. Target salt 2025: a global overview of national programs to encourage the food industry to reduce salt in foods. *Nutrients* **2014**, *6*, 3274-3287, doi:10.3390/nu6083274.
14. Kim, M.K.; Lee, K.G. Consumer awareness and interest toward sodium reduction trends in Korea. *Journal of food science* **2014**, *79*, S1416-1423, doi:10.1111/1750-3841.12503.
15. Losby, J.L.; Patel, D.; Schuldt, J.; Hunt, G.S.; Stracuzzi, J.C.; Johnston, Y. Sodium-reduction strategies for meals prepared for older adults. *Journal of public health management and practice : JPHMP* **2014**, *20*, S23-S30, doi:10.1097/PHH.0b013e3182a0e3ca.
16. Ma, G.X.; Shive, S.; Zhang, Y.; Aquilante, J.; Tan, Y.; Zhao, M.; Solomon, S.; Zhu, S.; Toubbeh, J.; Colby, L., et al. Knowledge, perceptions, and behaviors related to salt use among Philadelphia Chinese take-out restaurant owners and chefs. *Health promotion practice* **2014**, *15*, 638-645, doi:10.1177/1524839914538816.
17. Mezue, K. The increasing burden of hypertension in Nigeria - can a dietary salt reduction strategy change the trend? *Perspectives in public health* **2014**, *134*, 346-352, doi:10.1177/1757913913499658.
18. Marakis, G.; Tsigarida, E.; Mila, S.; Panagiotakos, D.B. Knowledge, attitudes and behaviour of Greek adults towards salt consumption: a Hellenic Food Authority project. *Public health nutrition* **2014**, *17*, 1877-1893, doi:10.1017/s1368980013002255.
19. Korošec, Ž.; Pravst, I. Assessing the average sodium content of prepacked foods with nutrition declarations: the importance of sales data. *Nutrients* **2014**, *6*, 3501-3515, doi:10.3390/nu6093501.

20. Vanderlee, L.; Hammond, D. Does nutrition information on menus impact food choice? Comparisons across two hospital cafeterias. *Public health nutrition* **2014**, *17*, 1393-1402, doi:10.1017/s136898001300164x.
21. Sookram, C.; Munodawafa, D.; Phori, P.M.; Varenne, B.; Alisalad, A. WHO's supported interventions on salt intake reduction in the sub-Saharan Africa region. *Cardiovascular diagnosis and therapy* **2015**, *5*, 186-190, doi:10.3978/j.issn.2223-3652.2015.04.04.
22. Enkhtungalag, B.; Batjargal, J.; Chimedsuren, O.; Tsogzolmaa, B.; Anderson, C.S.; Webster, J. Developing a national salt reduction strategy for Mongolia. *Cardiovascular diagnosis and therapy* **2015**, *5*, 229-237, doi:10.3978/j.issn.2223-3652.2015.04.11.
23. Kloss, L.; Meyer, J.D.; Graeve, L.; Vetter, W. Sodium intake and its reduction by food reformulation in the European Union — A review. *NFS Journal* **2015**, *1*, 9-19, doi:10.1016/j.nfs.2015.03.001.
24. Trieu, K.; Neal, B.; Hawkes, C.; Dunford, E.; Campbell, N.; Rodriguez-Fernandez, R.; Legetic, B.; McLaren, L.; Barberio, A.; Webster, J. Salt Reduction Initiatives around the World - A Systematic Review of Progress towards the Global Target. *PLoS One* **2015**, *10*, e0130247, doi:10.1371/journal.pone.0130247.
25. Lee, J.; Park, S. Consumer attitudes, barriers, and meal satisfaction associated with sodium-reduced meal intake at worksite cafeterias. *Nutr Res Pract* **2015**, *9*, 644-649, doi:10.4162/nrp.2015.9.6.644.
26. Webster, J.; Trieu, K.; Dunford, E.; Nowson, C.; Jolly, K.-A.; Greenland, R.; Reimers, J.; Bolam, B. Salt reduction in Australia: from advocacy to action. *Cardiovascular diagnosis and therapy* **2015**, *5*, 207-218, doi:10.3978/j.issn.2223-3652.2015.04.02.
27. Hendriksen, M.A.H.; Verkaik-Kloosterman, J.; Noort, M.W.; van Raaij, J.M.A. Nutritional impact of sodium reduction strategies on sodium intake from processed foods. *European journal of clinical nutrition* **2015**, *69*, 805-810, doi:10.1038/ejcn.2015.15.
28. Hendriksen, M.A.H.; van Raaij, J.M.A.; Geleijnse, J.M.; Breda, J.; Boshuizen, H.C. Health Gain by Salt Reduction in Europe: A Modelling Study. *PLOS ONE* **2015**, *10*, e0118873, doi:10.1371/journal.pone.0118873.
29. Do, H.T.; Santos, J.A.; Trieu, K.; Petersen, K.; Le, M.B.; Lai, D.T.; Bauman, A.; Webster, J. Effectiveness of a Communication for Behavioral Impact (COMBI) Intervention to Reduce Salt Intake in a Vietnamese Province Based on Estimations From Spot Urine Samples. *Journal of clinical hypertension (Greenwich, Conn.)* **2016**, *18*, 1135-1142, doi:10.1111/jch.12884.
30. Land, M.-A.; Wu, J.H.Y.; Selwyn, A.; Crino, M.; Woodward, M.; Chalmers, J.; Webster, J.; Nowson, C.; Jeffery, P.; Smith, W., et al. Effects of a community-based salt reduction program in a regional Australian population. *BMC Public Health* **2016**, *16*, 388, doi:10.1186/s12889-016-3064-3.
31. Takada, T.; Imamoto, M.; Fukuma, S.; Yamamoto, Y.; Sasaki, S.; Uchida, M.; Miura, Y.; Shimizu, S.; Nihata, K.; Fukuhara, S. Effect of cooking classes for housewives on salt reduction in family members: a cluster randomized controlled trial. *Public health* **2016**, *140*, 144-150, doi:10.1016/j.puhe.2016.07.005.
32. Regan, Á.; Shan, C.L.; Wall, P.; McConnon, Á. Perspectives of the public on reducing population salt intake in Ireland. *Public health nutrition* **2016**, *19*, 1327-1335, doi:10.1017/s1368980015002530.
33. Quilez, J.; Salas-Salvado, J. The feasibility and acceptability of reducing salt in partially baked bread: a Spanish case study. *Public health nutrition* **2016**, *19*, 983-987, doi:10.1017/s1368980015000944.
34. Lee, J.; Park, S. Management of Sodium-reduced Meals at Worksite Cafeterias: Perceptions, Practices, Barriers, and Needs among Food Service Personnel. *Osong public health and research perspectives* **2016**, *7*, 119-126, doi:10.1016/j.phrp.2015.12.011.
35. McLaren, L.; Sumar, N.; Barberio, A.M.; Trieu, K.; Lorenzetti, D.L.; Tarasuk, V.; Webster, J.; Campbell, N.R.C. Population‐level interventions in government jurisdictions for dietary sodium reduction. *Cochrane Database of Systematic Reviews* **2016**, 10.1002/14651858.CD010166.pub2, doi:10.1002/14651858.CD010166.pub2.
36. Curtis, C.J.; Clapp, J.; Niederman, S.A.; Ng, S.W.; Angell, S.Y. US Food Industry Progress During the National Salt Reduction Initiative: 2009-2014. *American journal of public health* **2016**, *106*, 1815-1819, doi:10.2105/ajph.2016.303397.
37. Park, S.; Lee, J. 'When operating a cafeteria, sales come before nutrition' - finding barriers and facilitators to serving reduced-sodium meals in worksite cafeterias. *Public health nutrition* **2016**, *19*, 1506-1516, doi:10.1017/s1368980015002827.
38. Wang, M.; Moran, A.E.; Liu, J.; Coxson, P.G.; Penko, J.; Goldman, L.; Bibbins-Domingo, K.; Zhao, D. Projected Impact of Salt Restriction on Prevention of Cardiovascular Disease in China: A Modeling Study. *PLoS One* **2016**, *11*, e0146820, doi:10.1371/journal.pone.0146820.

39. van Buren, L.; Dötsch-Klerk, M.; Seewi, G.; Newson, R.S. Dietary Impact of Adding Potassium Chloride to Foods as a Sodium Reduction Technique. *Nutrients* **2016**, *8*, 235, doi:10.3390/nu8040235.
40. Grimes, C.A.; Kelley, S.-J.; Stanley, S.; Bolam, B.; Webster, J.; Khokhar, D.; Nowson, C.A. Knowledge, attitudes and behaviours related to dietary salt among adults in the state of Victoria, Australia 2015. *BMC Public Health* **2017**, *17*, 532, doi:10.1186/s12889-017-4451-0.
41. Brooks, C.J.; Barrett, J.; Daly, J.; Lee, R.; Blanding, N.; McHugh, A.; Williams, D.; Gortmaker, S. A Community-Level Sodium Reduction Intervention, Boston, 2013-2015. *American journal of public health* **2017**, *107*, 1951-1957, doi:10.2105/AJPH.2017.304070.
42. Johnson, C.; Santos, J.A.; McKenzie, B.; Thout, S.R.; Trieu, K.; McLean, R.; Petersen, K.S.; Campbell, N.R.C.; Webster, J. The Science of Salt: A regularly updated systematic review of the implementation of salt reduction interventions (September 2016-February 2017). *Journal of clinical hypertension (Greenwich, Conn.)* **2017**, *19*, 928-938, doi:10.1111/jch.13099.
43. Nilson, E.A.F.; Spaniol, A.M.; Gonçalves, V.S.S.; Oliveira, M.L.; Campbell, N.; L'Abbé, M.; Jaime, P.C. The impact of voluntary targets on the sodium content of processed foods in Brazil, 2011–2013. *The Journal of Clinical Hypertension* **2017**, *19*, 939-945, doi:10.1111/jch.13044.
44. Pravst, I.; Lavriša, Ž.; Kušar, A.; Miklavec, K.; Žmitek, K. Changes in Average Sodium Content of Prepacked Foods in Slovenia during 2011-2015. *Nutrients* **2017**, *9*, doi:10.3390/nu9090952.
45. Okuda, M.; Asakura, K.; Sasaki, S. Placing Salt/Soy Sauce at Dining Tables and Out-Of-Home Behavior Are Related to Urinary Sodium Excretion in Japanese Secondary School Students. *Nutrients* **2017**, *9*, 1290, doi:10.3390/nu9121290.
46. Harnack, L.J.; Cogswell, M.E.; Shikany, J.M.; Gardner, C.D.; Gillespie, C.; Loria, C.M.; Zhou, X.; Yuan, K.; Steffen, L.M. Sources of Sodium in US Adults From 3 Geographic Regions. *Circulation* **2017**, *135*, 1775-1783, doi:10.1161/circulationaha.116.024446.
47. Inguglia, E.S.; Zhang, Z.; Tiwari, B.K.; Kerry, J.P.; Burgess, C.M. Salt reduction strategies in processed meat products – A review. *Trends in Food Science & Technology* **2017**, *59*, 70-78, doi:10.1016/j.tifs.2016.10.016.
48. Hendriksen, M.A.H.; Geleijnse, J.M.; van Raaij, J.M.A.; Cappuccio, F.P.; Cobiac, L.C.; Scarborough, P.; Nusselder, W.J.; Jaccard, A.; Boshuizen, H.C. Identification of differences in health impact modelling of salt reduction. *PLOS ONE* **2017**, *12*, e0186760, doi:10.1371/journal.pone.0186760.
49. Temme, E.H.M.; Hendriksen, M.A.H.; Milder, I.E.J.; Toxopeus, I.B.; Westenbrink, S.; Brants, H.A.M.; van der, A.D. Salt Reductions in Some Foods in The Netherlands: Monitoring of Food Composition and Salt Intake. *Nutrients* **2017**, *9*, doi:10.3390/nu9070791.
50. Lacey, M.; Chandra, S.; Tzianetas, R.; Arcand, J. Evaluation of actions, barriers, and facilitators to reducing dietary sodium in health care institutions. *Food science & nutrition* **2018**, *6*, 2337-2343, doi:10.1002/fsn3.814.
51. Gupta, P.; Mohan, S.; Johnson, C.; Garg, V.; Thout, S.R.; Shivashankar, R.; Krishnan, A.; Neal, B.; Prabhakaran, D. Stakeholders' perceptions regarding a salt reduction strategy for India: Findings from qualitative research. *PLOS ONE* **2018**, *13*, e0201707, doi:10.1371/journal.pone.0201707.
52. Trieu, K.; Ieremia, M.; Santos, J.; Neal, B.; Woodward, M.; Moodie, M.; Bell, C.; Snowdon, W.; Faumuina, T.; Webster, J. Effects of a nationwide strategy to reduce salt intake in Samoa. *J Hypertens* **2018**, *36*, 188-198, doi:10.1097/hjh.0000000000001505.
53. Levi, R.; Probst, Y.; Crino, M.; Dunford, E. Evaluation of Australian soup manufacturer compliance with national sodium reduction targets. *Nutrition & dietetics: the journal of the Dietitians Association of Australia* **2018**, *75*, 200-205, doi:10.1111/1747-0080.12392.
54. Zhang, P.; He, F.J.; Li, Y.; Ma, J.; Wu, J.; Wang, H.; Li, Y.; Han, J.; Luo, R.; Wang, Y., et al. Salt reduction in China: from evidence to action. *The Lancet* **2018**, *392*, S29, doi:10.1016/S0140-6736(18)32658-8.
55. Ahn, S.-H.; Kwon, J.S.; Kim, K.; Lee, Y.; Kim, H.-K. Current status, perception and practicability of restaurant staffs related to reducing sodium use in Seongnam, Korea. *jnh* **2019**, *52*, 475-487, doi:10.4163/jnh.2019.52.5.475.
56. Sparks, E.; Paterson, K.; Santos, J.A.; Trieu, K.; Hinge, N.; Tarivonda, L.; Snowdon, W.; Johnson, C.; Webster, J. Salt-Related Knowledge, Attitudes, and Behaviors on Efate Island, Vanuatu. *Int J Environ Res Public Health* **2019**, *16*, 1027, doi:10.3390/ijerph16061027.
57. Dodd, R.; Ramanathan, S.; Angell, B.; Peiris, D.; Joshi, R.; Searles, A.; Webster, J. Strengthening and measuring research impact in global health: lessons from applying the FAIT framework. *Health Research Policy and Systems* **2019**, *17*, 48, doi:10.1186/s12961-019-0451-0.

58. Beer-Borst, S.; Hayoz, S.; Eisenblätter, J.; Jent, S.; Siegenthaler, S.; Strazzullo, P.; Luta, X. RE-AIM evaluation of a one-year trial of a combined educational and environmental workplace intervention to lower salt intake in Switzerland. *Prev Med Rep* **2019**, *16*, 100982-100982, doi:10.1016/j.pmedr.2019.100982.
59. Arcand, J.; Blanco-Metzler, A.; Benavides Aguilar, K.; L'Abbe, M.R.; Legetic, B. Sodium Levels in Packaged Foods Sold in 14 Latin American and Caribbean Countries: A Food Label Analysis. *Nutrients* **2019**, *11*, doi:10.3390/nu11020369.
60. Yang, Y.X.; Gao, C.; Wang, Z.; Wang, Y.Y.; Lai, J.Q.; Ding, G.G. [Introduction to Guidelines for Salt Reduction in Chinese Food Industry]. *Zhonghua Yu Fang Yi Xue Za Zhi* **2019**, *53*, 549-552, doi:10.3760/cma.j.issn.0253-9624.2019.06.002.
61. Park, H.-K.; Lee, Y.; Kang, B.-W.; Kwon, K.-i.; Kim, J.-W.; Kwon, O.-S.; Cobb, L.K.; Campbell, N.R.C.; Blakeman, D.E.; Kim, C.-i. Progress on sodium reduction in South Korea. *BMJ Global Health* **2020**, *5*, e002028, doi:10.1136/bmjgh-2019-002028.
62. Bolton, K.A.; Webster, J.; Dunford, E.K.; Jan, S.; Woodward, M.; Bolam, B.; Neal, B.; Trieu, K.; Reimers, J.; Armstrong, S., et al. Sources of dietary sodium and implications for a statewide salt reduction initiative in Victoria, Australia. *The British journal of nutrition* **2020**, *123*, 1165-1175, doi:10.1017/s000711452000032x.
63. Dunford, E.K.; Poti, J.M. Simulating the impact of sodium reduction from packaged foods on population sodium intake in US adults and children. *Public health nutrition* **2020**, *23*, 488-495, doi:10.1017/s1368980019002696.
64. Sloan, A.A.; Keane, T.; Pettie, J.R.; Bhuiya, A.R.; Taylor, L.N.; Bates, M.; Bernard, S.; Akinleye, F.; Gilchrist, S. Mapping and Analysis of US State and Urban Local Sodium Reduction Laws. *Journal of Public Health Management and Practice* **2020**, *26*.
65. Doggui, R.; Al-Jawaldeh, H.; Al-Jawaldeh, A. Trend of Iodine Status in the Eastern Mediterranean Region and Impact of the Universal Salt Iodization Programs: a Narrative Review. *Biological trace element research* **2020**, *198*, 390-402, doi:10.1007/s12011-020-02083-1.
